# Aripiprazole-induced reversible myopia in a patient with tourette syndrome

**DOI:** 10.1007/s10072-025-08496-x

**Published:** 2025-09-16

**Authors:** Andrea E. Cavanna, Elisa Capriolo, Virginia Caimi, Gabriele Arienti, Anna Riva, Renata Nacinovich, Stefano Seri

**Affiliations:** 1 Department of Neuropsychiatry, BSMHFT and School of Medical Sciences, College of Medicine and Health, University of Birmingham, Birmingham, UK; 2 Sobell Department of Motor Neuroscience and Movement Disorders, Institute of Neurology, University College London, London, UK; 3 School of Health and Life Sciences, Aston Institute of Health and Neurodevelopment, Aston University, Birmingham, UK; 4 School of Medicine and Surgery, University of Milano-Bicocca, Milan, Italy; 5 Department of Child Neuropsychiatry, Fondazione IRCCS San Gerardo dei Tintori, Monza, Italy

**Keywords:** Aripiprazole, Myopia, Tics, Tourette syndrome

## Abstract

**Background:**

Myopia has been reported as a rare adverse effect of aripiprazole, a third-generation antidopaminergic medication used for the treatment of different neuropsychiatric conditions, including psychotic disorders, affective disorders, and obsessive-compulsive disorder.

**Case description:**

We document the rare case of a 29-year-old woman diagnosed with a neurodevelopmental tic disorder (Tourette syndrome) who developed bilateral myopia while taking aripiprazole 15 mg daily as a first-line anti-tic agent. Her myopia completely regressed following a decrease in the dose of aripiprazole from 15 mg to 10 mg daily.

**Discussion:**

To date, a total of 11 cases presenting with aripiprazole-induced reversible myopia (9 females, age range 19–34 years) have been reported. In addition to broadening the spectrum of the treated conditions, our case report raises the possibility of dose-dependent mechanisms underlying the development of myopia, at least in selected cases.

## Introduction

Aripiprazole is a third-generation antidopaminergic medication whose efficacy has been primarily attributed to a combination of partial agonism at the human dopamine D2 and serotonin 5-HT1A receptors, coupled with antagonism at the serotonin 5-HT2A receptors [1]. The most common indications are psychotic disorders, affective disorders (depression, bipolar disorder), obsessive-compulsive disorder, and tic disorders [2].

Aripiprazole is characterised by a relatively favourable tolerability profile, especially in terms of the extrapyramidal and metabolic adverse effects that are commonly associated with other antidopaminergic agents. In particular, the use of aripiprazole has been associated with reduced serum prolactin levels and is less likely to result in QTc interval prolongation [2]. The most common adverse effects of aripiprazole include insomnia, akathisia, headache, nausea, vomiting, weight gain, and somnolence. Visual defects, such as transient myopia and diplopia, are rare occurrences, documented as scattered case reports in the medical literature [1–10]. We present here the first case of a patient with a tic disorder (Tourette syndrome, TS) who developed aripiprazole-induced reversible myopia.

## Case report

A.B., a 29-year-old woman with a longstanding history of tics, was referred to the specialist Tourette syndrome Clinic, Department of Neuropsychiatry, BSMHFT and University of Birmingham (United Kingdom), for the assessment and management of her symptoms. The age at tic onset was 7, and the first tic to be noticed was eye blinking. She subsequently developed multiple motor tics, including frowning, raising eyebrows, eye rolling, nose twitching, nostril flaring, upper lip movements, mouth pouting, lip licking, swallowing, breath holding, jaw movements, platysma tightening, head drooping, neck extending, neck stretching, torso tensing, and hip movements. In terms of phonic tics, she reported mild throat clearing only. There were no pali/echo/coprophenomena or other socially inappropriate behaviours. Her tics were characteristically preceded by premonitory urges and could be voluntarily suppressed at the expense of mounting inner tension. She reported her tics as being exacerbated by anxiety and alleviated by physical activity and active concentration. In addition to anxiety (for which she was taking Sertraline 150 mg daily), she presented with mild obsessional thoughts, but no tic-related obsessive compulsive behaviours. There was no childhood history of attention-deficit and hyperactivity disorder. There was no previous history of visual dysfunction. She reported a family history of both tic disorders (paternal uncle) and anxiety disorders (maternal cousin).

On neurological examination, there was evidence of multiple motor tics, mainly affecting her face and neck. Both her clinical history and presentation were consistent with a relatively uncomplicated form of TS, characterised by predominantly motor tics. She scored 56/100 on the Tourette syndrome Diagnostic Confidence Index (slightly below the average scores reported at specialist clinics) and 42/100 on the Yale Global Tic Severity Scale, indicating moderate tic severity. Underlying anxiety was identified as her main tic-exacerbating factor.

In consideration of the impact of her multiple motor tics on her health-related quality of life, she was prescribed aripiprazole as a first-line anti-tic agent. Aripiprazole was gradually titrated up to the dose of 15 mg daily with beneficial effects on tic severity, however after about one month she developed bilateral myopia of mild-to-moderate severity (−3.0 diopters). Within two weeks of decreasing the dose of aripiprazole from 15 mg daily to 10 mg daily, her bilateral myopia resolved. She did not require either further decreases or switching to different anti-tic pharmacotherapy.

## Discussion

To the best of our knowledge, a total of ten cases of aripiprazole-related myopia [1–10] have been reported in the existing literature (Table 1).

**Table 1:** Case studies of aripiprazole-induced reversible myopia

Case report	Age, sex	Diagnosis	Aripiprazole dose (mg/day)	Myopia development (days)	Myopia severity (diopters)	Intervention	Myopia regression (days)
Kaya et al., [3]	21 F	Bipolar disorder	15	7	RE: −7.0 LE: −8.0	Discontinuation	10
Selvi et al., [4]	19 F	OCD	10	15	RE: −4.0 LE: −4.5	Discontinuation	10
Nair et al., [5]	33 M	Psychosis	15	30	RE: −3.0 LE: −3.0	Discontinuation	10
Karadağ et al., [6]	30 F	Psychosis	20	5	RE: −3.0 LE: −3.0	Discontinuation + Switch to paliperidone	N/A
Güneş et al., [7]	34 F	Depression + OCD	10	3	RE: 0 LE: −2.0	Discontinuation	3–4
Praveen Kumar et al., [8]	22 F	Psychosis	20	3	RE: −3.5 LE: −3.5	Discontinuation	14
Abreu and Pinheiro, [9]	21 M	Psychosis	20	14	RE: N/A LE: N/A	Discontinuation + Switch to paliperidone	8–10
Cumurcu et al., [10]	21 F	Depression	15	3	RE: −6.0 LE: −6.0	Discontinuation	30
Bulgu and Genc, [1]	34 F	Depression	N/A	7	RE: −2.0 LE: −2.0	Discontinuation	14
Kavak Sinanoğlu and Yavrum, [2]	27 F	Psychosis	30	14	RE: −2.5 LE: −2.5	Discontinuation + Switch to paliperidone	10
Present case	29 F	Tourette syndrome	15	30	RE: −3.0 LE: −3.0	Dose decrease	14

**Abbreviations.** *LE* left eye, *N/A* not available, *OCD* obsessive-compulsive disorder, *RE* right eye

Eight out of the ten patients were females, with ages ranging from 19 to 34 years. Aripiprazole-induced myopia was bilateral in all cases but one. The degree of myopia ranged between − 2.0 diopters (mild severity) and − 6.0 diopters (moderate severity). In the reported cases, aripiprazole-induced myopia developed over 3–30 days and regressed 3–30 days following aripiprazole discontinuation (plus switching to paliperidone in three cases). The most common indication was psychosis (*n* = 5), followed by affective disorders (*n* = 4) and obsessive-compulsive disorder (*n* = 2). We provided the first report of reversible bilateral myopia in a patient treated with aripiprazole for a different condition, namely a neurodevelopmental tic disorder (TS). Based on her clinical history, our patient reported a Naranjo Adverse Drug Reaction Probability Scale score of 7, consistent with a probable association between her myopia and the administered pharmacotherapy. Our case is also the first report of myopia resolution following aripiprazole dose decrease (from 15 mg daily to 10 mg daily), rather than discontinuation. In only two out of the ten reports myopia developed at a dose of aripiprazole that was lower than 15 mg daily (10 mg daily in both cases). Taken together, these findings might call into question previous suggestions about the absence of a dose-dependent association between aripiprazole and the development of myopia [2].

To date, aripiprazole-induced reversible myopia has been reported exclusively in young adults (no reports of patients older than 34 years) and predominantly in females (9/11 reports). Little is known about the physiopathology of this condition. The proposed mechanisms include aripiprazole-induced ciliary body and choroidal effusion resulting in swelling, anterior displacement of the ciliary processes, narrowing of the ciliary sulcus, and anterior displacement of the iris and lens [2]. It is also possible that aripiprazole causes myopia by directly entering the lens and triggering a swelling process by osmosis [2]. The possibility of a dose-dependent effect might pave the way for further research avenues into the complex processes underlying this rare, albeit clinically significant, occurrence.

Antidopaminergic agents including atypical antipsychotics have the potential to induce diverse unwanted ocular effects, ranging from transient mydriasis to problems with accommodation and angle-closure glaucoma in susceptible patients. Both retinal effects and ocular dystonia appear to be proportional to the total amount of drug used over a prolonged period of time [11].

In conclusion, myopia as a rare and possibly dose-dependent adverse effect of aripiprazole has been reported to date in patients with psychosis, affective disorders, obsessive-compulsive disorder, and tic disorders. Doses of aripiprazole ranging between 10 mg and 30 mg daily have been associated with this reversible adverse event. The time-course of myopia development can range from 3 to 30 days, with a similar timeframe for symptom regression following aripiprazole discontinuation or – as in our case – dose decrease. Further research is needed to establish the exact nature and prevalence of this adverse effect of aripiprazole, as well as to shed light on its underlying pathophysiological mechanisms across different clinical populations.
